# Mortality in Different Mountain Sports Activities Primarily Practiced in the Winter Season—A Narrative Review

**DOI:** 10.3390/ijerph17010259

**Published:** 2019-12-30

**Authors:** Martin Niedermeier, Hannes Gatterer, Elena Pocecco, Anika Frühauf, Martin Faulhaber, Verena Menz, Johannes Burtscher, Markus Posch, Gerhard Ruedl, Martin Burtscher

**Affiliations:** 1 Department of Sport Science, University of Innsbruck, 6020 Innsbruck, Austria; martin.niedermeier@uibk.ac.at (M.N.); elenapocecco@yahoo.it (E.P.); anika.fruehauf@uibk.ac.at (A.F.); Martin.Faulhaber@uibk.ac.at (M.F.); verena.menz@uibk.ac.at (V.M.); Markus.Posch@uibk.ac.at (M.P.); gerhard.ruedl@uibk.ac.at (G.R.); 2 Institute of Mountain Emergency Medicine, EURAC Research, 39100 Bolzano, Italy; hannes.gatterer@eurac.edu; 3 Center for Teaching Methodology, Pedagogical University Tyrol, 6020 Innsbruck, Austria; 4 Austrian Society for Alpine and High-Altitude Medicine, 6020 Innsbruck, Austria; 5 Laboratory of Molecular and Chemical Biology of Neurodegeneration, École Polytechnique Fédérale de Lausanne, 1015 Lausanne, Switzerland; johannes.burtscher@epfl.ch

**Keywords:** mountain sports, risk, mortality, death risk, alpine skiing, snowboarding, cross-country skiing, ski touring, sledging

## Abstract

Annually, millions of people engage in mountain sports activities all over the world. These activities are associated with health benefits, but concurrently with a risk for injury and death. Knowledge on death rates is considered important for the categorization of high-risk sports in literature and for the development of effective preventive measures. The death risk has been reported to vary across different mountain sports primarily practiced in the summer season. To complete the spectrum, the aim of the present review is to compare mortality rates across different mountain sports activities primarily practiced in winter. A comprehensive literature search was performed on the death risk (mortality) during such activities, i.e., alpine (downhill) skiing, snowboarding, cross-country skiing, ski touring, and sledging. With the exception of ski touring (4.4 deaths per 1 million exposure days), the mortality risk was low across different winter sports, with small activity-specific variation (0.3–0.8 deaths per 1 million exposure days). Traumatic (e.g., falls) and non-traumatic (e.g., cardiac death) incidents and avalanche burial in ski tourers were the predominant causes of death. Preventive measures include the improvement of sport-specific skills and fitness, the use of protective gear, well-targeted and intensive training programs concerning avalanche hazards, and sports-medical counseling for elderly and those with pre-existing diseases.

## 1. Introduction

Mountainous areas such as the Alps attract millions of international tourists and sports practitioners. Skier days (understood as initial entry to a ski resort, irrespective of the activity) are often used as a metric to indicate the popularity of winter sport regions [1]. For Austria, 54.6 million skier days were recorded for the season 2017/2018 according to the Professional Association of Austrian Cable Cars [2]. The number of skier days was constantly high since 2000/2001, with figures ranging between 43.7 million (2006/07) and 56.8 million skier days (2008/09) [2]. For Switzerland, 23.4 million skier days were recorded for the season 2017/2018 with 24.5 million skier days per year as a ten-year average [3]. Alpine (downhill) skiing as the predominantly conducted mountain sports activity in the winter season [4] and snowboarding are included in these figures. Additional frequently conducted winter sports activities usually not covered in these figures include cross-country skiing, ski touring or sledging. Solely with regard to alpine (downhill) skiing and snowboarding, about 400 million skier days have been estimated across 80 countries annually [5].

Relevant health benefits associated with leisure-time exercise are well accepted [6,7,8], although outdoor activities also bear an inherent risk of injury and even death [9]. Knowledge on the mortality risk associated with various types of winter sports is important to assess the hazardous nature of a certain type of sport. Such information might be helpful for participants and researchers with regard to two aspects: Firstly, mortality rates are a solid basis to define and categorize sports as high-risk/extreme sports in the literature [10]. Secondly, establishing the extent of the problem (i.e., mortality rate) is considered the basis for the development of effective preventive measures [11,12,13]. On this basis, risk factors, causes, and mechanisms of fatalities may be identified before focusing on preventive aspects. Furthermore, activity-specific mortality rates enable specifically focusing on activities with high risk for fatalities.

While we previously reviewed mortality rates in mountain sports activities primarily practiced in the summer season [14], this review aims to compare mortality rates in mountain sports activities primarily practiced during the winter season. Where available, potential risk factors and causes of deaths were reported and compared between these sports.

## 2. Materials and Methods

A nonsystematic literature search was conducted using various sources of information. Primarily, the following databases were screened for original articles or review articles using a cut-off month of August 2019: Pubmed/Medline, Web of Science, Science direct, Scopus, Sport Discus. We are not aware of a categorization of all mountain winter sports. Therefore, mountain sports were selected on the basis of injury/fatality statistics [15] and suggestions by the Austrian Alpine Association [16]. The following keywords were used in the literature search: death risk, mortality, alpine skiing, downhill skiing, snowboarding, cross-country skiing, ski touring, sledging, sledding, sleighing, and tobogganing. Reference lists of articles were also reviewed to ensure relevant studies were included. Furthermore, we used the unique database of the “Österreichisches Kuratorium für Alpine Sicherheit”. These data included all fatalities that occurred in the Austrian mountains from 1997 to 2018 [15,17]. More detailed information is provided in [18].

All relevant articles specifically for the mountain sports activities were reviewed by two authors responsible for each mountain sports activity: alpine (downhill) skiing and snowboarding (M.P. and G.R.), cross-country skiing (V.M. and M.B.), (alpine) ski touring (J.B. and M.B.), sledging (G.R. and M.P.), respectively. Data extraction was conducted by the first author mentioned for each mountain sports activity. Primarily, fatality numbers related to the population at risk and/or to the number of injuries/accidents, and/or specific information on exposure time were extracted from the selected studies and the injury/fatality statistics in the Austrian mountains [15,17]. Fatality rates were descriptively compared.

## 3. Results

The total number of studies and databases considered relevant for describing the mortality and potentially associated factors was *n* = 31 (alpine (downhill) skiing and snowboarding, *n* = 10; cross-country skiing, *n* = 6; (alpine) ski touring, *n* = 18; sledging, *n* = 5).

### 3.1. Alpine (Downhill) Skiers and Snowboarders

Alpine (downhill) skiing is a popular outdoor sports activity [19], and snowboarding has gained increasing popularity during the last three decades [20]. Both alpine skiing and snowboarding are primarily performed during the winter season on snow-covered slopes of mountainous areas [4]. Skiers and snowboarders use ski lifts and cable cars for the ascent, which is followed by downhill turns [21]. The majority of fatality statistics in skiing and snowboarding did not distinguish between skiers and snowboarders. In Austria, snowboarders accounted for less than 6% of all deaths between 2000 and 2018 [15,17]. Thereby, skiers and snowboarders were described collectively as ‘skiers’ in the present chapter.

Deaths during skiing can be of traumatic (e.g., collision with an object/person) or nontraumatic nature (e.g., cardiac death). From 1980 to 2001, a rising trend of deaths rates could be shown in a study by Xiang and Stallones (2003), who investigated mortality trends of skiing and recreational snow activities (cross-country skiing) over 21 ski seasons [22]. Death rates ranged from 0.53 (1981/82) to 1.88 (1986/87) per 1 million skier days [22]. In other reports, the rate of traumatic deaths among skiers has remained relatively stable over the past few decades, ranging from 0.37 [23] over 0.70 [24] to 0.75 deaths per 1 million skier days [25]. It has to be mentioned that some fatality rates only consider traumatic deaths on ski slopes [24,25]. In Austria, the overall incidence of both traumatic and nontraumatic deaths during the last two decades (2000–2018) was 0.77 deaths per 1 million skier days (Table 1), with an evaluated incidence of 0.35 nontraumatic deaths and 0.42 traumatic deaths per 1 million skier days [15,17]. There is no evidence to assume that fatality rates in alpine skiing in Austria have significantly changed in the past 20 years [15,17]. The majority (54.5%) of fatalities on Austrian slopes were traumatic deaths [15,17]. According to the majority of studies [22,23,25], the most common scenario of traumatic death in skiing is a collision with a solid object such as trees or rocks, or a collision with another skier (Table 2) and would be mostly avoidable by choosing a skiing velocity appropriate for individual skiing skills [5]. About 65.3% of all victims who died due to a traumatic event wore a ski helmet at the time of the accident. Only Bianchi and Brugger (2015) reported falls during skiing to be the primary cause of traumatic deaths on ski slopes [26]. In Austria, the most common cause of traumatic death on ski slopes was a collision with other skiers or solid objects (50.2%) [15,17]. Previous research has shown that males older than 32 years account for more than 80% of all traumatic deaths [22,23,25,26,27]. Confirming these findings, the majority of males (72.5%) who died on Austrian ski slopes were older than 32 years [15,17].

Sudden cardiac death (SCD) represents the leading nontraumatic death at altitude during leisure time activities, such as recreational skiing [9,27]. Confirming these results, Austrian data show SCD as the major cause of nontraumatic deaths (99%) over the past 19 years [15,17]. SCD risk increases with older age [27]. The majority of nontraumatic deaths (96%) were in males older than 34 years [9,27]. The risk to suffer from SCD during skiing was greatest at altitudes up to 2000 m (77.1% of all deaths) compared to altitudes over 2000 m above sea level [9,27]. Furthermore, SCD risk was highest on the first day at altitude compared to consecutive days [26]. Further potential risk factors for fatal cardiovascular events during skiing are prior myocardial infarction (OR: 92.8; 95% CI: 22.8–379.1), hypertension (OR: 9.0; 95% CI: 4.0–20.6), and known coronary artery disease (OR: 4.8; 95% CI: 1.1–21.2) [27].

### 3.2. Cross-Country Skiers

Cross-country skiing (or Nordic skiing) is performed on dedicated groomed trails, using lightweight skis, bindings and boots which allow free movement of the heel. Unlike alpine ski touring, cross-country ski bindings cannot be fixed. Within cross-country skiing, two main skiing styles can be distinguished: The classic style is characterized by moving the skis parallel to the direction of travel, whereas for the skating style, the skis are moved at an angle to the overall direction of travel [31]. Cross-country skiing is a very popular winter sport, not only in North and Central Europe, but also in countries with a cold climate such as Canada and the US [32]. Cross-country skiing is a high-intensity physical activity, demanding both the upper and the lower body, and is often performed at moderate altitude and in a cold environment [33].

In general, there is convincing evidence that long-term endurance training (i.e., cross-country skiing) has positive health benefits and is associated with a reduced risk of cardiovascular disease and all-cause mortality [34,35,36]. However, research suggests that vigorous physical activity may be a triggering factor for cardiovascular morbidity and mortality [28,36,37]. Moreover, long-term endurance-trained cross-country skiers may have an increased risk of atrial fibrillation, which may cause cardioembolic stroke [38,39]. There are only few data on fatalities in cross-country skiers. For the Vasaloppet, the largest cross-country ski race in the world [39], a mortality rate during skiing of 2.6 deaths per 1 million person-hours or one death per 53,700 starters was reported (Table 1) [28]. All deaths (13 deaths from 1970 to 2005) occurred in men 30–72 years old, and all but one death was caused by cardiovascular disease either due to myocarditis, hypertrophic cardiomyopathy or myocardial infarction [28]. The risk for sudden cardiac death in the overall population for males aged between 35 and 70 years is one sudden cardiac death per 3,370,000 h [9], equaling to 0.3 deaths per 1 million person-hours. For Austria, data on mortality in cross-country skiers is available from 1997 to 2017, where on average 3 deaths per year occurred [15,17]. Unfortunately, no information about the cause or mechanisms of deaths was reported specifically for cross-country skiing [15,17]. In Colorado, 100 deaths (90 males, 10 females) across 21 ski seasons were reported in cross-country skiers [22]. Deaths were mainly due to suffocation (67%) and hypothermia (10%), with avalanches being the underlying cause of death [22].

The causes of fatalities in cross-country skiing differs greatly between Europe and the US. The difference in the primary causes of death (sudden cardiac death vs. avalanches) may be due to the location where the sport is performed (Table 2). Typically, in Europe, cross-country skiing is performed on well-prepared and marked cross-country ski trails, whereas for Colorado, Xiang and Stallones (2003) reported cross-country skiing takes place wherever snow is available [22].

### 3.3. (Alpine) Ski Tourers

Alpine ski touring here is defined as a subdiscipline of ski mountaineering. Whereas ski mountaineering includes mountaineering activities using any type of ski, alpine ski touring is conducted using alpine skis (in contrast to cross-country). Ski bindings permit free moving heels during ascent, which can be fixed for descending (downhill skiing) [40,41]. During uphill climbing, climbing skins are mounted on the running surface of the skis and are removed for downhill skiing. Sometimes skis are carried or left at a certain place (ski depot) to overcome steep snow slopes, and rock and/or ice passages.

The risk of injury and mortality varies dramatically depending on the terrain (secured ski slopes, off-piste slopes, glaciers, and rock and ice slopes) where alpine ski touring is performed [42,43,44,45,46]. Studies from the Austrian Alps reported an annual death rate of 1.83 per 100,000 alpine ski tourers [47]. The traumatic death rate (in contrast to sudden non-traumatic deaths) was 1.57 per 100,000 ski tourers compared to 0.52, 2.62, and 6.6 per 100,000 alpine skiers, mountain hikers, and rock/ice climbers, respectively [42]. Fatal avalanche accidents were included in these figures and represented the most frequent cause of death (69%) in ski tourers [29,48]. The mortality rate was much higher in males (90%) than in females [29]. The number of sudden non-traumatic death (mostly sudden cardiac deaths) was low (<1.0/100,000) until the age of 59 years but then increased steeply to 10.4 per 100,000 ski tourers aged above 59 years, again exclusively in males [42]. An overall death risk of 4.4 per 1 million exposure days was calculated (Table 1) [29]. Surprisingly, this risk was 5.3 for members of alpine associations and 3.7 for non-members. Particularly the death risk due to avalanche burial was higher in members, whereas that due to falling and cardiac arrest was lower. When ski tourers use secured ski slopes, the death risk from snow avalanches is virtually zero, and falls or collisions with firm obstacles or other skiers [44] or cardiovascular events [9] are main causes of fatality. By contrast, in unsecured mountainous terrain (snow covered backcountry area), burial by snow avalanches represents the primary cause of death in the Alps, in Canada, and in the US (Table 2) [29,48,49,50]. This is not surprising due to the fact that the risk of avalanches is often extremely difficult to assess and the chance of survival rapidly decreases with time and depth of burial [51,52]. Paradoxically, a somewhat elevated training/touring experience seems to be associated with a higher risk of avalanche death. An about 2-fold higher death risk from avalanches was demonstrated for members of alpine associations, indicating an elevated risk in those with increased training level [29]. Early reports from Canada present similar findings [53]. High-risk groups were young (36 years, median), experienced male back-country skiers visiting ski areas with a known moderate to extreme avalanche hazard [53]. It has been speculated that the present education and training available to ski tourers is inappropriate and may provoke a false sense of security [29]. By contrast, the lower risk of fatal falls and sudden cardiac death in members of alpine association indicates beneficial effects of physical fitness and experience [29,54].

Whereas in steep rocky and icy terrain, falls are predominant causes of death, falls in a crevasse may occur on glaciers, in particular when not using ropes during both the ascent and descent (downhill skiing) [43]. In rare cases, ski tourers die from hypothermia [45]. As for mountain hikers and downhill skiers, the risk of sudden cardiac death steeply increases in males above the age of 34 years and in particular in those with pre-existing cardiovascular diseases and/or low fitness levels [9]. The number of subjects at risk, e.g., all ski tourers, is often unknown for the assessment of the mortality risk and therefore the mortality index (MI; number of fatalities per number of accidents) has been introduced. A recent study from France reported a MI of 8% for ski touring, which was lower than that for mountaineering in rocky or icy terrains (MI: 10%) or climbing a Via Ferrata (MI: 10%) but was higher than for hiking on a trail (MI: 4%) [55]. Long-term mortality recordings from the Austrian Alps do not show any change with time [15,17]. Thus, mortality may largely be associated with weather, snow, and avalanche conditions. The percentages of deaths during ski touring from 2000 to 2017 varied between 5 and 14% (mean: 8%) related to the total numbers of fatalities during mountain sports activities in Austria [15,17].

### 3.4. Sledgers

Recreational sledging (also referred to as sledding, sleighing or tobogganing) is a popular low-cost winter pastime in mountainous regions. Sledging contains an uphill part to a highpoint (e.g., by feet or by cable car) and a downhill part using different types of wooden sledges, toboggans or plastic bobsleighs on snow hills, ski slopes or dedicated sledging tracks [56,57]. In South Tyrol, the upper part of Italy, 1.4 million sledging days per season were estimated in 2011 on 145 sledging tracks [58]. In Switzerland, a total of 1.8 million hours per year with a mean of 2 h per day for sledging was calculated for 2014, resulting in an average of 900,000 sledging days per year [59].

In a 3-year study on sledging injuries treated in an Emergency Department in South Tyrol, three sledging-related fatalities were reported [56]. Between the years 2000 and 2017, 11 fatal sledging-related injuries were recorded in Switzerland [30]. Assuming 900,000 sledging days annually in Switzerland [59], a risk of 0.7 fatal injuries per 1 million days of exposure days per year can be calculated for recreational sledgers in Switzerland (Table 1). In Austria, 32 fatalities with a sledge involved were recorded between 1997 and 2017, indicating an average of less than 1.6 deaths per year due to sledging in the Austrian Alps [15,17].

To the best of our knowledge, no published data on risk factors for fatalities in sledging are available. However, personal communications with the Österreichisches Kuratorium für Alpine Sicherheit revealed the following details for the Austrian Alps: Data from 20 recorded sledging-related deaths between 2006 and 2018 revealed a mean age of 40.0 (SD: 20.1, range: 3–68) years and 75% males. In addition, 85% of all fatal injuries occurred during descent, 45% due to a collision with an obstacle, 50% on sledging tracks, and 35% on ski slopes. A head injury was the major cause of traumatic deaths (40% of all traumatic deaths, Table 2); with no victim wearing a helmet (only 3 persons of the 20 reported fatalities used a helmet). Furthermore, 25% of non-traumatic deaths due to a cardiac–vascular event was reported with 100% males and a mean age of 51.4 (SD: 18.2, range: 23–68) years.

Sledging-related injuries in a retrospective study were associated with male sex, a mean age of 38.1 (SD: 15.1, range: 18–79) years, a higher sledging frequency, higher skill level, and risky behavior [60]. At least male sex and risky behavior may represent risk factors also for fatalities.

## 4. Discussion

The main aim of this narrative review was to compare mortality rates in mountain sports activities primarily practiced during the winter season. Except for cross-country skiing, trauma-related deaths are prevalent in all winter mountain sports activities that have been addressed. The main preventive measure to reduce traumatic deaths is the use of protective gear like helmets [61,62]. The helmet rate is already above 90% in alpine skiing [63], but still low in other winter sports (e.g., 50% and below in sledging [60]). In addition to sledging, helmet use should be especially promoted in snowboarders younger than 30 years and older than 60 years of age [62]. Further preventive measures include increased awareness of potential risk factors, e.g., fall and collision hazards, avalanche danger, and freezing and hypothermia risk, and the selection of a downhill skiing and sledging velocity appropriate to the individual skill level [5,23]. For alpine ski touring conducted in free ski terrain, avalanches were the most frequent hazard; it is worth noting that there is some evidence that experienced individuals are at higher risk to be buried by an avalanche. Although ski tourers being alone did not show a higher risk propensity compared to persons in large groups [64], it is recommended to conduct ski tours with one or more partners to reduce fatalities connected with avalanche burial. Partner rescue is crucial for survival, since the highest survival rates of avalanche victims were found within the first 15 min after burial [65]. Well-targeted and intensive training programs concerning avalanche hazards seem urgently needed.

With the exception of sledging, non-traumatic (mostly cardiac) deaths are not uncommon in all other winter mountain sports addressed. Protective factors include adequate physical preparation (e.g., regular aerobic exercise and specifically high-intensity exercise performed more than once per week for alpine skiing [21]) and a high number of winter sport days per year [27]. Rest and/or moderate intensity exercise on the first day at altitude followed by a progressive increase in intensity on the following days might additionally contribute to the prevention of cardiac death [9]. Furthermore, high exercise intensities at the beginning of the sports activity should be avoided [28]. Males and persons older than 34 years and those with pre-existing cardiovascular diseases are considered as potential high-risk groups [9,27,28].

When comparing the death rates across different winter sports activities, it has to be noted that the assessment of the numbers of individuals at risk is eminently difficult. Probably the most valid estimation of death rate in the present review is the death rate for cross-country skiing, since all starters of the race were included in the evaluation [28]. However, the fact the death rate of cross-country skiing is derived from a mass sport race event limits its comparability to the other sports activities addressed in this review. For alpine skiing and snowboarding, data on skier days might provide a useful estimation for individuals at risk. However, alpine ski touring and sledging is usually self-organized and without an easy-to-screen proof for participation (e.g., overnight stay, ticket or summit attempts). Therefore, death rates in the present review are often based on rough estimations, which limits the validity of these figures.

This is one reason why we did not follow the approach of a systematic review or meta-analysis (e.g., Preferred Reporting Items for Systematic Reviews and Meta-Analyses (PRISMA) Statement [66]). Another reason is the limited number of scientific work on this topic, which makes a systematic approach including an adequate number of data for analysis problematic, if not unfeasible. The authors did their best to provide a balanced selection of available studies; however, a bias in study selection and assessment cannot be ruled out. To the best of our knowledge, this is the first attempt to compare the mortality risk during several winter sports activities, which may constitute a valuable basis for future studies.

## 5. Conclusions

When compared to mountain sports activities primarily conducted in the summer season, mortality risk varied less across different winter mountain sports. Mortality risk was lowest for cross-country skiing and highest for ski touring in the winter sports compared. However, it has to be mentioned that mortality risk in cross-country skiing was estimated from competition conditions, rendering comparison to other winter sports activities difficult (e.g., due to population differences). Except for ski touring, the predominant causes of death were traumatic and cardiac events. Preventive measures include the improvement of sport-specific skills and fitness, the use of protective gear, well-targeted and intensive training programs concerning avalanche hazards, and sports-medical counseling for elderly and those with pre-existing diseases.

## Figures and Tables

**Table 1:** Selected mortality rates during different sports activities in the mountains.

Mountain Sports Activity	Region/Mountain	Mortality Risk (Death Rate per 1,000,000 Exposure Days)	Time Period	Reference
Alpine (downhill) skiing and snowboarding	Austrian Alps	0.77	2000–2018	[15,17]
Cross-country skiing	Sweden Vasaloppet	0.26 ^a^	1970–2005	[28] ^a^
Alpine ski touring	Austrian Alps	4.4	1986–1995	[29]
Sledging	Switzerland	0.7	2000–2017	[30]

^a^ Based on an average race time of 8, 5, and 4 h for the 90, 45, and 30 km races, respectively.

**Table 2:** Main causes and risk factors in different mountain sports activities.

Mountain Sports Activity	Main Causes	Main Risk Factors
Alpine (downhill) skiing	Non-traumatic (mostly cardiac) death	Male sex
and snowboarding	Trauma-related death (collisions, falls)	Pre-existing cardiovascular diseases
		Higher age
		Lack of fitness
		Risky behavior
Cross-country skiing	Non-traumatic (mostly cardiac) death	Male sex
	Avalanches	Higher age
		Pre-existing diseases
Alpine ski touring	Avalanche burial	Male sex
	Trauma-related death (falls)	Lack of appropriate training
	Non-traumatic (mostly cardiac) death	Risky behavior
		Pre-existing diseases
		Lack of fitness
Sledging	Trauma-related death (collisions)	Lack of protective gear
		Lack of appropriate training
